# Research Participants’ Engagement and Retention in Digital Health Interventions Research: Protocol for Mixed Methods Systematic Review

**DOI:** 10.2196/65099

**Published:** 2025-01-03

**Authors:** Luciana Terceiro, Mudassir Imran Mustafa, Maria Hägglund, Anna Kharko

**Affiliations:** 1 Department of Women's and Children's Health CIRCLE - Complex Intervention Research in Health and Care Uppsala University Uppsala Sweden; 2 Department of Women's and Children's Health Participatory eHealth and Health Data Research Group Uppsala University Uppsala Sweden; 3 Medtech Science & Innovation Centre Uppsala University Hospital Uppsala Sweden; 4 School of Psychology, Faculty of Health University of Plymouth Plymouth United Kingdom

**Keywords:** clinical research informatics, participant engagement, participant retention, clinical research, mobile application, digital platforms, mobile phone

## Abstract

**Background:**

Digital health interventions have become increasingly popular in recent years, expanding the possibilities for treatment for various patient groups. In clinical research, while the design of the intervention receives close attention, challenges with research participant engagement and retention persist. This may be partially due to the use of digital health platforms, which may lack adequacy for participants.

**Objective:**

This systematic literature review aims to investigate the relationship between digital health platforms and participant engagement and retention in clinical research. It will map and analyze key definitions of engagement and retention, as well as identify design characteristics that influence them.

**Methods:**

We will carry out a mixed methods systematic literature review, analyzing qualitative and quantitative studies. The search strategy includes the electronic databases PubMed, IEEE Xplore, CINAHL, Scopus, Web of Science, APA PsycINFO, and the ACM Digital Library. The review will encompass studies published between January 2018 and June 2024. Criteria for inclusion will be the presence of digital health care interventions conducted through digital health platforms like websites, web and mobile apps used by patients, and informal caregivers as research participants. The main outcome will be a narrative analysis with key findings on the definitions of participant engagement and retention and critical factors that affect them. Quality assessment and appraisal will be done through the Mixed-Methods Assessment Tool. Data analysis and synthesis will follow the PRISMA (Preferred Reporting Items for Systematic Reviews and Meta-Analyses) 2020 flow diagram. Quantitative data will be qualified and integrated into qualitative data, which will be analyzed using thematic analysis and synthesis.

**Results:**

The study expects to map and summarize critical definitions of participant engagement and retention, and the characteristics of digital health platforms that influence them. The systematic review is expected to be completed in June 2025.

**Conclusions:**

This systematic review will contribute to the growing discussion on how the design of digital health intervention platforms can promote participant engagement and retention in clinical research.

**Trial Registration:**

PROSPERO CRD42024561650; https://www.crd.york.ac.uk/prospero/display_record.php?RecordID=561650

**International Registered Report Identifier (IRRID):**

PRR1-10.2196/65099

## Introduction

In 2022, over 100,000 health care mobile apps were available in Apple and Google app stores combined [1]. Digital health care has transformed care delivery through a diverse fleet of technologies, from mobile apps and wearable devices to biosensors and the Internet of Things [2]. It offers a myriad of innovative ways to provide treatments, monitor health conditions, assist, and empower patients with diverse needs to be more in charge of their health, and enable health care professionals to deliver better service [3]. Following the expansion of the digital health care range, digital health (DH) interventions have also increased exponentially (Textbox 1). DH interventions are interventions delivered through digital technology for the treatment or management of physical or mental conditions [4].

**Key concepts.**

**Digital health intervention**

- Interventions are delivered through digital technologies such as smartphones, websites, wearables, video games, or text messaging [2,5,6]. A digital health intervention offers guidance, information, and support for a diversity of physical or mental health conditions through a digital platform. Also commonly referred to as health informatics or eHealth interventions [7]; they are designed to help people avoid, recover from, or cope with disease and disability or to improve the quality and safety of health care [8], for example, as self-help or self-guided eHealth interventions [9,10].

**Digital health platforms**

- Websites, web-based or mobile apps used to access digital health interventions.

**Digital clinical research**

- Clinical research is conducted through digital health platforms. It may include digital health interventions, digital data collection, and electronic Case Report Forms, among other resources. Only digital clinical research encompassing digital health interventions will be considered for this study.

**Research participant**

- Recipients of intervention; for example, patients or informal caregivers.

**Participant engagement**

- Length and depth of participant’s involvement with the digital health intervention.

**Participant retention**

- Duration and continuity of the participant’s involvement with the digital health intervention.

**Figure 1:**
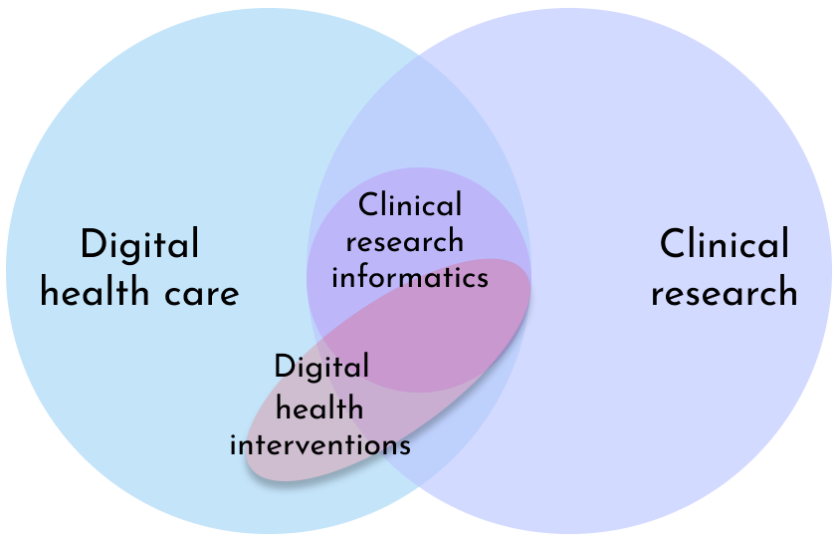
Digital health interventions are part of general digital health care and clinical research. The latter can be delivered through clinical research informatics or commercial health platforms and software.

Through online treatments, DH interventions promise to improve health care, enhancing accessibility, effectiveness, and personalization [2,11]. DH interventions are available in commercial applications, as easily accessible health care, and as part of clinical research (Figure 1). When conducted as part of clinical research, they share the same benefits as general digital health care. DH interventions also allow for the development of effective treatments for more patients [11], are more community-inclusive [12], decrease health disparities [13], and improve study generalizability and validity [14,15]. Successful clinical research generates evidence that, in turn, promotes health care improvements [16].

In clinical research, DH interventions are designed following the principles of clinical research informatics (CRI) [4]. CRI is the use of informatics principles and techniques to conduct clinical research [4]. DH interventions have the potential to accelerate the process from initial research to “real world” outcomes, contributing to increasing scale and distribution, cost and resource optimization, and facilitating financial auditing processes [12,16-18]. These principles could also be applied to DH interventions.

Both engagement and retention of clinical research participants are crucial intervention research outcomes, but the concepts have varying definitions. Frequently, different terms for engagement are used interchangeably, like involvement, participation, acceptability, and completion rates, among others. Engagement can be described as the extent and manner in which people actively use a resource [19]. Perski et al [20] define engagement as two main concepts: (1) a subjective experience, meaning a state of focus and interest with a temporal dissociation, and (2) a behavior, described as usage over time. It is often connected to concepts like adherence, duration, and frequency that can be quantified through concrete measurements like opening or using a mobile app, frequency of times, or the duration of the use [21]. Participant retention, in turn, refers to the proportion of recruited participants who remained in the study until its end and at an optimal proportion that does not compromise the study’s validity [22,23].

In DH intervention research, both engagement and retention can vary considerably. Intervention dropout from internet-based treatment for psychological disorders, for example, fluctuates between 30% and 50% [24]. While poor engagement or retention may be due to the intervention quality or outside factors, an often-overlooked component is the design of DH platforms. Appraising the DH platforms’ design choices and how they impact participant engagement and retention could help make DH interventions better. In this context, there is an untapped opportunity to explore the practical factors that affect intervention success. This systematic literature review will focus on DH platforms’ design choices concerning engagement and retention and their relationship with research participants’ behavior.

The relationship between DH platforms and research participants is receiving growing interest, as evidenced by the increase in research. Studies have examined participant engagement and retention across various settings, for example, mHealth or web-based platforms [21,25], focusing on particular patient groups, for example, older adults with dementia or digital mental health interventions [26-29].

Still, a comprehensive literature review on the relationship between DH interventions and participant engagement and retention in digital clinical research has yet to be conducted. This review aims to fill the gap by studying DH platforms that have been used in digital clinical research. We will map the various definitions that capture the engagement and retention of research participants in DH interventions. We will further identify platform design factors and features that hinder or promote participants’ engagement and retention. The key concepts relevant to the review are defined in Textbox 1.

## Methods

This systematic review was submitted for registration with the International Prospective Register of Systematic Reviews (PROSPERO) on June 8, 2024 (CRD42024561650), to avoid bias in conducting and reporting findings. According to the study’s progress, amendments will be made if necessary [30].

### Review Question

The review question was elaborated using the Population-Exposure-Outcome (PEO) statement, as outlined in Textbox 2. We chose to apply the PEO as it is regarded as the more appropriate approach for qualitative inquiries [31]. It is also more suitable for the definition of associations between particular exposures and factors and related outcomes [32]. The overall review question is: What factors and aspects promote research participants’ engagement with and retention in DH interventions in digital clinical research? This was further broken down into 2 specific research questions:

Research question 1: How are engagement and retention of research participants defined in DH interventions conducted for clinical research?

Research question 2: What user interface elements, interaction design, and platform characteristics influence research participants’ engagement and retention in DH interventions?

**Population-exposure-outcome structure.**

Population-exposure-outcome element and description

**Population**

Research participants who are patients, informal or family caregivers.

**Exposure**

User interface, interaction elements, and platform characteristics of digital health interventions conducted in clinical research.

**Outcomes**

Engagement and retention of research participants.

### Methodology Choice Rationale

The choice to perform a mixed methods systematic literature review is due to the number of individual studies that have already been conducted in digital health care and DH interventions, providing substantial evidence for the review.

The methodology selected for this systematic literature review is the mixed methods systematic review (MMSR) [33]. It is a standard approach that allows to systematically combine qualitative and quantitative data [34]. By integrating the findings of effectiveness (quantitative data) with findings on participants’ experiences (qualitative data), MMSR offers a comprehensive evaluation with balanced data insights [35].

We plan to carry out the MMSR as we expect both data types to be prevalent in the reviewed studies. By including both data types, we will adopt a holistic approach to defining engagement and retention. For instance, qualitative data can shed light on the context, patient and informal caregivers’ experiences, and barriers to engagement and retention, which quantitative data alone may not fully capture.

### Search Strategy

We will analyze studies that (1) offered a DH intervention; (2) used a DH platform component, such as a mobile app, website, or text-messaging process; (3) collected engagement and retention-related measurements–quantitative, qualitative, or both; and (4) presented a digital interface to the research participants—patients, and informal caregivers—to interact with the DH intervention. The DH platforms can be designed specifically for clinical research or not. Commercially available health applications will be considered if they are used for clinical research purposes.

The search strategy for this systematic literature review was developed in collaboration with Görel Sundström, a librarian from Uppsala University, and the researchers involved in this study, LT, AK, MH, and MIM.

The PEO statement was used to construct the search strategy (Table 1). The keywords refinement process involved different approaches: tests conducted by the librarian, consultation of referenced articles to analyze the keywords they used, and expert reviews conducted by the research team. The keywords selection process was performed to ensure the search would capture studies using various terminologies to address the same research questions (Multimedia Appendix 1).

**Table 1:** Preliminary Web of Science search strategy (to be adapted for the other databases).

Search number			Database search algorithm
**User engagement, user retention, and metrics**			
	1	(“active user*” OR Attrition OR “Click rate” OR “Completion rate*” OR “Frequency of use” OR “Follow up” OR Login OR “log in” OR “Returning user*” OR “Session duration” OR “Sign in” OR “Study complet*” OR “Time spent” OR usage OR “User actions” OR “Use Rate*” OR “User metric*” OR “user session*”)	
	2	((Caregiver* OR “Healthy Volunteer*” OR “Research Subject*” OR participant* OR patient* OR subject* OR user*) NEAR/3 (accept* OR activit* OR adher* OR attitude* OR barrier* OR challeng* OR complian* OR discontinu* OR Disengagement* OR Dropout* OR Efficien* OR Effectiveness OR engag* OR evalutation* OR experience* OR Finish* OR involvement* OR interaction* OR obstacle* OR participation* OR perception* OR perspective* OR retention* OR satisf* OR visit* OR view*))	
	3	1 OR 2	
**Clinical research informatics and digital care**			
	4	(“Clinical informat*” OR “Clinical research informat*” OR “Clinical trials informatic*” OR CRI OR “digital care” OR eHealth OR e-health OR etherap* OR “e-Mental health” OR “Health informati*” OR iCBT OR “Internet Cognitive Behavioral Treatment*” OR “medical informatics*” OR mHealth OR m-health OR mtherap* OR m-therap* OR “Online Clinical Trial*” OR telerehabilitation)	
	5	((“clinical research” OR “clinical trial*” OR “medical research” OR health OR intervention* OR psychotherap* OR therap* OR “self-help program*” OR treatment*) NEAR/3 (Computer* OR cyber OR Digital OR electronic OR informatics OR Internet OR Mobile OR Online OR Smartphone OR “Technology Based” OR “Web based”))	
	6	4 OR 5	
**Design and aspects of software or digital platform**			
	7	(“Interaction design*” OR Interface OR Usability OR “User centered design*” OR “Visual design*”)	
**Combining all topics**			
	8	3 AND 6 AND 7	

The search will be conducted across a range of electronic databases: PubMed, IEEE Xplore, CINAHL, Scopus, Web of Science, APA PsycINFO, and the ACM Digital Library. These databases are chosen based on their relevance to the research topic and their widespread use in academic and research communities.

In addition to the electronic database searches, the research team will use additional search methods to identify potential studies that may not be captured through the database searches. It includes hand search, which involves manually scanning relevant journals; back-forward citation tracking, where we examine the references of identified articles; and reference checking to ensure no valuable sources are overlooked during the review process.

This systematic literature review will not involve collecting sensitive personal data.

### Study Selection Criteria

The PEO statement was used to outline the eligibility criteria for study inclusion and exclusion, delineating them by population, exposure, and outcomes (Table 2).

**Table 2:** Population-exposure-outcome inclusion and exclusion criteria.

PEO^a^	Inclusion criteria	Exclusion criteria
Population	Research participants, study participants, patients, informal caregivers, carers, caregivers, and users. No exclusion based on age or gender.	Researchers, physicians, doctors, nurses, social care workers, social workers, dentists, and health care professionals.
Exposure or environment	User interface and interaction design of DH platforms.	Engagement and experience related to the intervention or treatment itself. Experience with content quality (text and multimedia content).
Outcomes	Engagement and retention to the study.	Efficacy of the treatment, efficacy related to the intervention or treatment itself.
Study methods	Qualitative methods, quantitative methods, mixed methods.	Reviews (systematic, scoping, meta-analysis, etc)
Publication types	Formally published peer-reviewed journal articles, conference papers.	Grey literature, opinion pieces, protocols, reviews
Geographical considerations	Initially not limited to any geographical area.	

^a^PEO: Population-exposure-outcome.

#### Types of Studies

##### Qualitative Studies

Qualitative interviews, focus group discussions, usability studies, participatory research, participatory design, case studies, grounded theory research, thematic and content analysis of textual data, phenomenological studies, narrative research, and ethnographic observations.

##### Quantitative Studies

Randomized controlled trials, cohort studies, longitudinal studies, experimental studies, case-control studies, cross-sectional studies, and observational studies.

##### Mixed Methods Studies

Studies integrating qualitative and quantitative data collection and analysis methods within a single research design, encompassing but not restricted to convergent design, sequential explanatory design, and sequential exploratory design.

In the case of studies addressing the same DH intervention and cohort of individuals, only the study with more detailed data regarding engagement and retention-based measurements will be considered, unless the studies present different aspects of the 2 mentioned subjects.

Studies such as gray literature, editorials, letters, opinion papers, and theses and dissertations will be excluded.

#### Time Period

The study will consider articles published from January 2018 to June 2024. This 7-year publication window was chosen because of the rapid evolution in the technology and health informatics domain. The timeframe also covers DH intervention platforms developed before and after the COVID-19 pandemic [36].

#### Language

Due to resource constraints, the study will exclusively include articles published in English. The research team acknowledges that this approach limits the inclusion of studies performed in different parts of the world and published in other languages.

### Study Screening

First, a search conducted by an independent librarian will identify potentially eligible studies based on predefined keywords that consider the inclusion and exclusion criteria. The results from this initial search will undergo deduplication: Duplicates will be identified and removed using EndNote (version 21; Clarivate), using the Bramer et al [37] guidelines. The remaining studies will then be imported into Covidence [38]. There, data will be screened in two steps: (1) title and abstract screening, and (2) full-text screening. During the first step, at least 2 reviewers will independently review the titles and abstracts, blinded to the authors’ names, and each other’s review decisions (ie, double-blinded) [38]. After, the potential articles will have their full text screened, filtered, and categorized according to the predefined inclusion and exclusion criteria. In the event of disagreements between the 2 reviewers, at either stage of the review process, a third reviewer (LT) will be consulted to reach a consensus.

### Data Extraction

The data extraction will use a standardized data extraction form elaborated by the research team. The form is designed to capture study information such as (1) identification: study ID, authors, year, country, publication type, and analysis type (qualitative, quantitative, and mixed-methods); (2) characteristics: research participants’ characteristics, age, sample size, intervention description, digital platform or software, and the medium used; (3) results: engagement and completion measurements, results and findings presented in qualitative and quantitative data, and measurement tools.

Other information may be added as the research team considers it relevant to the analysis. One reviewer will independently extract the data, and a second reviewer will check it for accuracy and completion. The extracted information will be organized in a previously formatted table in Microsoft Excel. Qualitative data regarding results and findings will also be collected. The qualitative data will be analyzed using NVivo (version 14; Lumivero) afterward.

### Quality Assessment and Appraisal

The study plans to use the mixed-methods assessment tool (MMAT) for quality assessment and critical appraisal [39]. MMAT provides a systematic approach to assessing quality criteria on a variety of study designs, such as qualitative studies, quantitative randomized controlled trials, quantitative nonrandomized, quantitative descriptive, and mixed-methods studies [40]. Using MMAT, we will evaluate the studies’ clarity of the research question, appropriateness of the study design, data collection methods, data analysis, and interpretation of results.

### Data Analysis and Synthesis

**Figure 2:**
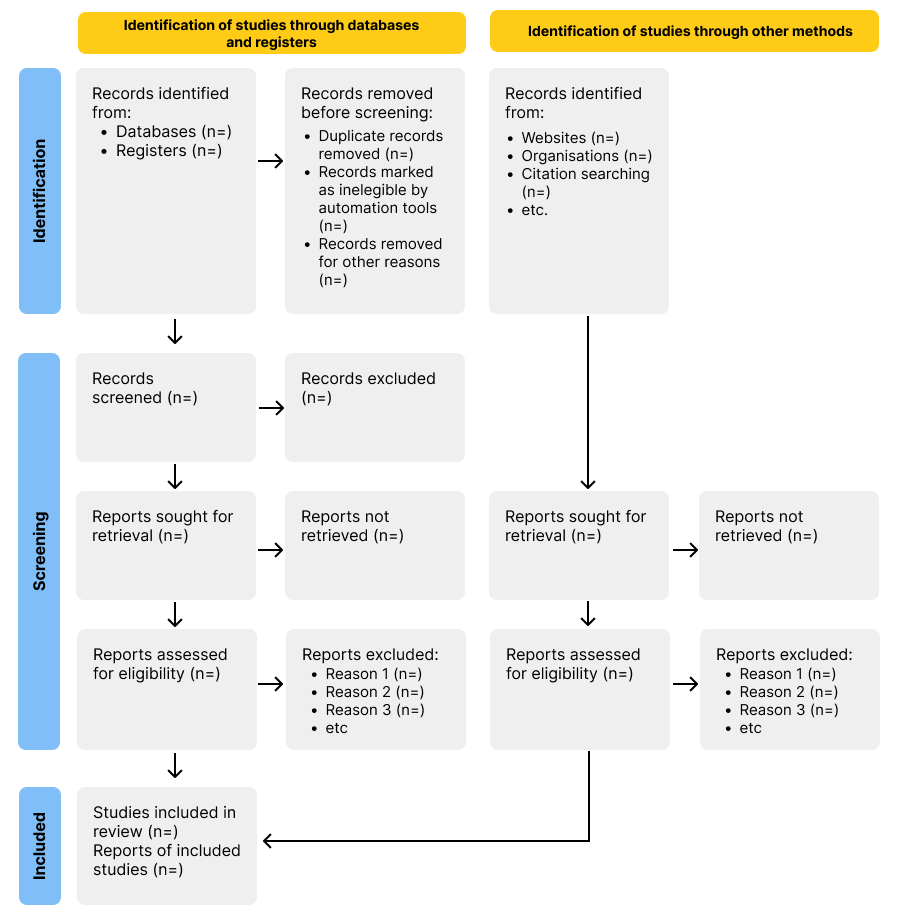
PRISMA (Preferred Reporting Items for Systematic Reviews and Meta-Analyses) flow diagram.

The study selection procedure will be visualized through the PRISMA 2020 flow diagram, as seen in Figure 2 [41].

Data related to the identification of a given study and its characteristics will be organized in summary tables. As the review question stipulates the inclusion of a wide range of research designs, including qualitative and quantitative designs, the main results from our investigation will be organized into major themes and subthemes, and key findings on terms will also be presented. If significant differences and patterns arise, such as those related to health conditions, age, or digital literacy, thematic clusters for analysis will be delineated. Since the data will potentially arise from diverse study designs, quantitative data will be submitted to a data transformation process to be qualitized to be converted into qualitative data in the form of themes and categories and afterward summarized in a narrative synthesis to allow further integration with qualitative data [35,42,43]. Once qualitative and quantitative data are integrated, they will be compiled through a thematic analysis in order to identify the main concepts regarding engagement and retention. The codes for this investigation will be developed by one of the reviewers and checked by at least 1 member of the research team. The codes will be built using the Persuasive System Design framework developed by Oinas-Kukkonen and Harjumaa as a basis [44]. Kelders et al [25] have already applied this framework in the digital health area. A total of 2 independent reviewers will conduct the coding, and discrepancies and new codes will be discussed between the 2 reviewers. If no agreement is reached, a third reviewer will be consulted to reach a consensus. Afterward, the major themes and subthemes will be summarized in a narrative synthesis. One of the authors will compose the narrative synthesis, and a second author will assess and provide appraisal.

### Dissemination Strategy

The results of this study will be disseminated as a scientific publication in a peer-reviewed journal and presented at conferences. Plain-language summaries will also be produced to share in various channels, such as social media, ResearchGate, and technology and health care websites.

### Ethical Considerations

According to the Ethical Review Data (2003:460) by the Swedish Ethical Review Authority, ethical approval will not be required for this research.

## Results

As of June 2024, the literature review has conducted 2 pilot searches to test and refine keywords and verify the initial quality of results. The results are expected to be published as a systematic literature review and submitted for publication in June 2025.

## Discussion

### Principal Findings

In light of the potential benefits of technology in clinical research, DH intervention design demands further investigation, to mediate the relationship between research participants and the technology. As highlighted by Johnson [45], connected technologies have provided many new opportunities in clinical research in recent years, such as increasing research awareness, recruitment options, and delivering interventions and treatments. Achieving a high rate of participation required to ensure the quality of an investigation is still a challenge. To meet these opportunities, CRI researchers and developers becoming more aware of the importance of developing adequate software for research participants to expand intervention. Although a “user-centric” approach has increased through participant-centered initiatives, digital clinical research is still on the journey to find ways to reduce the time and labor requirements that hinder participant involvement [14,46]. Offering a proper setting to a plurality of participants is fundamental to guaranteeing clinical research quality; otherwise, CRI risks increasing health care inequalities and disparities. DH interventions that do not consider socioeconomic factors such as financial situation, race, ethnicity, age, education, and digital literacy present higher chances of producing intervention-generated inequalities, increasing the digital divide, and may only benefit the already more advantaged populations [6,9,47].

Intervention researchers have long experimented with strategies for engagement and retention. Intervention factors like acceptability and feasibility of devices and technology, system usability, visual design, content, and adaption to literacy levels have been found to affect participant behaviors. These factors commonly influence access conditions by minimizing attrition but do not necessarily guarantee engagement, retention, and adherence. Importantly, engagement and retention may be promoted by factors that pertain to the particular characteristics of the digital platforms and software. Here are included platform usability and design, but also and others are research-based strategies, such as compensation, incentives, or rewards [21]. Interaction features can also incentivize participant’s engagement and retention. These could be in the form of (1) gamification, (2) reminders or notifications, (3) social support provided within the DH intervention, (4) personalization, and (5) content tailored to participants’ physical and cognitive abilities [21,25,26]. Understanding how platform design choices interact with participant behavior in DH interventions has become a crucial consideration for intervention research.

### Limitations

Summarizing the key DH platform factors that affect participant engagement and retention given the variability of intervention designs, target participant groups and DH platform mediums may be challenging. Given the heterogeneity of the reviewed studies, we may have to focus only on some participant populations or DH platforms or include only broad trends in the narrative synthesis. Preliminary research, however, showed that concepts like personalization and fit to participants’ conditions and needs are commonly important design factors, as discussed in previous literature [11].

### Implications

We foresee that this review will serve as a useful resource to those developing DH interventions, but may not be versed in DH platform design. By summarizing key platform design characteristics that affect participant behavior on the platform and, by proxy, the intervention, the review will be particularly relevant to intervention researchers.

### Conclusions

Systematic reviews are considered one of the most informative sources of research evidence and have supported decision-making in health care in recent decades [40,48]. Acknowledging the relevance of this resource, this review aims to contribute to the growing field of digital clinical research and patient-centered design, providing a comprehensive reference for developing more engaging and effective digital platforms and software for clinical research.

## Appendix

Multimedia Appendix 1 String searches.

## Abbreviations

CRI: clinical research informatics
DH: digital health
MMAT: mixed-methods assessment tool
MMSR: mixed-methods systematic review
PEO: population-exposure-outcomes
PRISMA: Preferred Reporting Items for Systematic Reviews and Meta-Analyses
PROSPERO: Prospective Register of Systematic Reviews

## Data Availability

Data sharing is not applicable to this article as no datasets were generated or analyzed during this study.
