# Supplementary material for: Research Participants’ Engagement and Retention in Digital Health Interventions Research: Protocol for Mixed Methods Systematic Review
Source: JMIR Res Protoc. 2025 Jan 3;14:e65099. doi: 10.2196/65099 (PMC11748419; doi:10.2196/65099)
Supplement: Multimedia Appendix 1 [file resprot_v14i1e65099_app1.pdf]

Multimedia Appendix 1

ACM, 2024-09-04

Search no	Search terms
1	"active user*" OR "Attrition" OR "Click rate" OR "Completion rate*" OR "Frequency of use" OR "Follow up" OR "Login" OR "log in" OR "Session duration" OR "Sign in" OR "Study complet*" OR "Time spent" OR "usage" OR "User actions" OR "Use Rate*" OR "User metric*" OR "user session*"
2	"Caregiver*" OR "Healthy Volunteer*" OR "Research Subject*" OR "participant*" OR "patient*" OR "user*"
3	"accept*" OR "activit*" OR "adher*" OR "attitude*" OR "barrier*" OR "challeng*" OR "complian*" OR "discontinu*" OR "Disengag*" OR "Dropout*" OR "Efficien*" OR "Effectiveness" OR "engag*" OR "evalutation*" OR "experience*" OR "Finish*" OR "involvement*" OR "interaction*" OR "obstacle*" OR "participation*" OR "perception*" OR "perspective*" OR "retention*" OR "satisf*" OR "visit*" OR "view*"
4	2 AND 3
5	1 OR 4
6	"Clinical informat*" OR "Clinical research informat*" OR "Clinical trials informatic*" OR "CRI" OR "digital care" OR "eHealth" OR "e-health" OR "etherap*" OR "e-Mental health" OR "Health informati*" OR "iCBT" OR "medical informatic*" OR "mHealth" OR "m-health" OR "mtherap*" OR "m-therap*" OR "Online Clinical Trial*"
7	"clinical research" OR "clinical trial*" OR "medical research" OR "health" OR "intervention*" OR "psychotherap*" OR "therap*" OR "self-help program*" OR "treatment*"
8	"Computer*" OR "cyber" OR "Digital" OR "electronic" OR "informatics" OR "Internet" OR "Mobile" OR "Online" OR "Smartphone" OR "Technology Based" OR "Web based"
9	7 AND 8
10	6 OR 9
11	"design feature*" OR "interaction design*" OR "Interface" OR "User centered design*" OR "visual design*" OR "usability" OR "graphic design*"
	5 AND 10 AND 11
	Filter 5 year

IEEE, 2024-09-04

Search no	Search terms
1	"All Metadata":"active user" OR "All Metadata":Attrition OR "All Metadata":"Click rate" OR "All Metadata":"Completion rate*" OR "All Metadata":"Frequency of use" OR "All Metadata":"Follow up" OR "All Metadata":Login OR "All Metadata":"log in" OR "All Metadata":"Returning user" OR "All Metadata":"Session duration" OR "All Metadata":"Sign in" OR "All Metadata":"Study completion" OR "All Metadata":"Time spent" OR "All Metadata":usage OR "All Metadata":"User actions" OR "All Metadata":"Use Rate" OR "All Metadata":"User metric" OR "All Metadata":"user session"
2	("All Metadata":Caregiver OR "All Metadata":"Healthy Volunteer" OR "All Metadata":"Research Subject" OR "All Metadata":participant OR "All Metadata":patient OR "All Metadata":user OR "All Metadata":Caregivers OR "All Metadata":"Healthy Volunteers" OR "All Metadata":"Research Subjects" OR "All Metadata":participants OR "All Metadata":patients OR "All Metadata":users) NEAR/3 ("All Metadata":acceptance OR "All Metadata":accepting OR "All Metadata":activit* OR "All Metadata":adhering OR "All Metadata":adherence OR "All Metadata":attitude OR "All Metadata":barrier OR "All Metadata":attitudes OR "All Metadata":barriers OR "All Metadata":challenges OR "All Metadata":compliance OR "All Metadata":discontinue OR "All Metadata":Disengaging OR "All Metadata":Dropout OR "All Metadata":Efficience OR "All Metadata":Effectiveness OR "All Metadata":engagaging OR "All Metadata":evalutation OR "All Metadata":experience OR "All Metadata":experiences OR "All Metadata":Finish OR "All Metadata":finishing OR "All Metadata":involvement OR "All Metadata":interaction OR "All Metadata":interactions OR "All Metadata":obstacle OR "All Metadata":obstacles OR "All Metadata":participation OR "All Metadata":perception OR "All Metadata":perspective OR "All Metadata":perceptions OR "All Metadata":perspectives OR "All Metadata":retention OR "All Metadata":satisf* OR "All Metadata":visit OR "All Metadata":visits OR "All Metadata":view)
3	1 OR 2
4	("All Metadata":"Clinical informatics" OR "All Metadata":"Clinical research informatics" OR "All Metadata":"Clinical trials informatics" OR "All Metadata":CRI OR "All Metadata":"digital care" OR "All Metadata":eHealth OR "All Metadata":e-health OR "All Metadata":etherapy OR "All Metadata":etherapies OR "All Metadata":"e-Mental health" OR "All Metadata":"Health informatics" OR "All Metadata":iCBT OR "All Metadata":"Internet Cognitive Behavioral Treatment" OR "All Metadata":"medical informatics" OR "All Metadata":mHealth OR "All Metadata":m-health OR "All Metadata":mtherapy OR "All Metadata":m-therapy OR "All Metadata":"Online Clinical Trial" OR "All Metadata":mtherapies OR "All Metadata":m-therapies OR "All Metadata":"Online Clinical Trials")
5	(("All Metadata":"clinical research" OR "All Metadata":"clinical trial" OR "All Metadata":"clinical trials" OR "All Metadata":"medical research" OR "All Metadata":health OR "All Metadata":intervention OR "All Metadata":interventions OR "All Metadata":psychotherapy OR "All Metadata":therapy OR "All Metadata":"self-help program" OR "All Metadata":treatment OR "All Metadata":psychotherapies OR "All Metadata":therapies OR "All Metadata":"self-help programs" OR "All Metadata":treatments) NEAR/3 ("All Metadata":Computer* OR "All Metadata":cyber OR "All Metadata":Digital OR "All Metadata":electronic OR "All Metadata":informatics OR "All Metadata":Internet OR "All Metadata":Mobile OR "All Metadata":Online OR "All Metadata":Smartphone OR "All Metadata":"Technology Based" OR "All Metadata":"Web based"))
6	4 OR 5
7	("All Metadata":"design feature" OR "All Metadata":"interaction design" OR "All Metadata":Interface OR "All Metadata":"User centered design" OR "All Metadata":"visual design" OR "All Metadata":usability OR "All Metadata":"graphic design" OR "All Metadata":"graphical design" OR "All Metadata":"design features" OR "All Metadata":"interaction designs" OR "All Metadata":"User centered designs" OR "All Metadata":"visual designs" OR "All Metadata":"graphic designs" OR "All Metadata":"graphical designs")
8	3 AND 6 AND 7
9	Filter 2019-2024

PsycInfo, 2024-08-29

Search no	Search terms
1	DE "Health Attitudes" OR DE "Health Attitude Measures"
2	"active user*" OR Attrition OR "Click rate" OR "Completion rate*" OR "Frequency of use" OR "Follow up" OR Login OR "log in" OR "Returning user*" OR "Session duration" OR "Sign in" OR "Study complet*" OR "Time spent" OR usage OR "User actions" OR "Use Rate*" OR "User metric*" OR "user session*"
3	DE "Patients" OR DE "Caregivers" OR DE "Experimental Subjects"
4	Caregiver* OR "Healthy Volunteer*" OR "Research Subject*" OR participant* OR patient* OR user*
5	3 OR 4
6	accept* OR activit* OR adher* OR attitude* OR barrier* OR challeng* OR complian* OR discontinu* OR Disengag* OR Dropout* OR Efficien* OR Effectiveness OR engag* OR evalutation* OR experience* OR Finish* OR involvement* OR interaction* OR obstacle* OR participation* OR perception* OR perspective* OR retention* OR satisf* OR visit* OR view*
7	5 AND 6
8	1 OR 2 OR 7
9	DE "Health Information Technology"
10	"Clinical informat*" OR "Clinical research informat*" OR "Clinical trials informatic*" OR CRI OR "digital care" OR eHealth OR e-health OR etherap* OR "e-Mental health" OR "Health informati*" OR "Internet Cognitive Behavioral Treatment*" OR iCBT OR "medical informatic*" OR mHealth OR m-health OR mtherap* OR m-therap* OR "Online Clinical Trial*"
11	9 OR 10
12	DE "Clinical Trials" OR DE "Health Care Delivery"
13	"clinical research" OR "clinical trial*" OR "medical research" OR health OR intervention* OR psychotherap* OR therap* OR "self-help program*" OR treatment*
14	12 OR 13
15	DE "Communications Media" OR DE "Digital Media" OR DE "Information and Communication Technology" OR DE "Mobile Applications" OR DE "Smartphones"
16	Computer* OR cyber OR Digital OR electronic OR informatics OR Internet OR Mobile OR Online OR Smartphone OR "Technology Based" OR "Web based"
17	15 OR 16
18	14 AND 17
19	11 OR 18
20	"design feature*" OR "interaction design*" OR Interface OR "User centered design*" OR "visual* design*" OR usability OR "graphic* design*"
21	8 AND 19 AND 20
22	Filter 5 year

PubMed, 2024-08-30

Search no	Search terms
1	Attitude to health [MeSH] OR Retention in Care[MeSH] OR Evaluation Studies as Topic [MeSH]
2	"active user*" OR Attrition OR "Click rate" OR "Completion rate*" OR "Frequency of use" OR "Follow up" OR Login OR "log in" OR "Session duration" OR "Sign in" OR "Study complet*" OR "Time spent" OR usage OR "User actions" OR "Use Rate*" OR "User metric*" OR "user session*"
3	"Patients"[Mesh] OR "Caregivers"[Mesh] OR "Research Subjects"[Mesh]
4	Caregiver* OR "Healthy Volunteer*" OR "Research Subject*" OR participant* OR patient* OR user*
5	3 OR 4
6	accept* OR activit* OR adher* OR attitude* OR barrier* OR challeng* OR complian* OR discontinu* OR Disengag* OR Dropout* OR Efficien* OR Effectiveness OR engag* OR evalutation* OR experience* OR Finish* OR involvement* OR interaction* OR obstacle* OR participation* OR perception* OR perspective* OR retention* OR satisf* OR visit* OR view*
7	5 AND 6
8	1 OR 2 OR 7
9	"Medical Informatics Applications"[Mesh:NoExp] OR "Medical Informatics"[Mesh:NoExp]
10	"Clinical informat*" OR "Clinical research informat*" OR "Clinical trials informatic*" OR CRI OR "digital care" OR eHealth OR e-health OR etherap* OR "e-Mental health" OR "Health informati*" OR iCBT OR "medical informatic*" OR mHealth OR m-health OR mtherap* OR m-therap* OR "Online Clinical Trial*"
11	9 OR 10
12	Biomedical Research [MeSH] OR "clinical trials as topic"[MeSH Terms] OR Research Design[MeSH] OR Delivery of Health Care[MeSH]
13	"clinical research" OR "clinical trial*" OR "medical research" OR health OR intervention* OR psychotherap* OR therap* OR "self-help program*" OR treatment*
14	12 OR 13
15	"Computer Communication Networks"[Mesh] OR "Digital Technology"[Mesh] OR "Information Technology"[Mesh] OR Mobile Applications [MeSH] OR "Smartphone"[Mesh]
16	Computer* OR cyber OR Digital OR electronic OR informatics OR Internet OR Mobile OR Online OR Smartphone OR "Technology Based" OR "Web based"
17	15 OR 16
18	14 AND 17
19	11 OR 18
20	"user-computer interface"[MeSH Terms] OR "Universal Design"[Mesh]
21	"design feature*" OR "interaction design*" OR Interface OR "User centered design*" OR "visual* design*" OR usability OR "graphic* design*"
22	20 OR 21
23	8 AND 19 AND 22
24	Filter 5 year

Scopus, 2024-08-30

Search no	Search terms
1	"active user*" OR Attrition OR "Click rate" OR "Completion rate*" OR "Frequency of use" OR "Follow up" OR Login OR "log in" OR "Returning user*" OR "Session duration" OR "Sign in" OR "Study complet*" OR "Time spent" OR usage OR "User actions" OR "Use Rate*" OR "User metric*" OR "user session*"<br>(Ti-Abs-Key)
2	((Caregiver* OR "Healthy Volunteer*" OR "Research Subject*" OR participant* OR patient* OR user*) W/3 (accept* OR activit* OR adher* OR attitude* OR barrier* OR challeng* OR complian* OR discontinu* OR Disengag* OR Dropout* OR Efficien* OR Effectiveness OR engag* OR evalutation* OR experience* OR Finish* OR involvement* OR interaction* OR obstacle* OR participation* OR perception* OR perspective* OR retention* OR satisf* OR visit* OR view*))<br>(Ti-Abs-Key)
3	1 OR 2
4	("Clinical informat*" OR "Clinical research informat*" OR "Clinical trials informatic*" OR CRI OR "digital care" OR eHealth OR e-health OR etherap* OR "e-Mental health" OR "Health informati*" OR iCBT OR "Internet Cognitive Behavioral Treatment*" OR "medical informatic*" OR mHealth OR m-health OR mtherap* OR m-therap* OR "Online Clinical Trial*")<br>(Ti-Abs-Key)
5	(("clinical research" OR "clinical trial*" OR "medical research" OR health OR intervention* OR psychotherap* OR therap* OR "self-help program*" OR treatment*) W/3 (Computer* OR cyber OR Digital OR electronic OR informatics OR Internet OR Mobile OR Online OR Smartphone OR "Technology Based" OR "Web based"))<br>(Ti-Abs-Key)
6	4 OR 5
7	("design feature*" OR "interaction design*" OR Interface OR "User centered design*" OR "visual* design*" OR usability OR "graphic* design*")<br>(Ti-Abs-Key)
8	3 AND 6 AND 7
9	Filter 2019-2024

Web of Science, 2024-08-30

Search no	Search terms
1	TS=("active user*" OR Attrition OR "Click rate" OR "Completion rate*" OR "Frequency of use" OR "Follow up" OR Login OR "log in" OR "Returning user*" OR "Session duration" OR "Sign in" OR "Study complet*" OR "Time spent" OR usage OR "User actions" OR "Use Rate*" OR "User metric*" OR "user session*")
2	TS=((Caregiver* OR "Healthy Volunteer*" OR "Research Subject*" OR participant* OR patient* OR user*) NEAR/3 (accept* OR activit* OR adher* OR attitude* OR barrier* OR challeng* OR complian* OR discontinu* OR Disengag* OR Dropout* OR Efficien* OR Effectiveness OR engag* OR evalutation* OR experience* OR Finish* OR involvement* OR interaction* OR obstacle* OR participation* OR perception* OR perspective* OR retention* OR satisf* OR visit* OR view*))
3	1 OR 2
4	TS=("Clinical informat*" OR "Clinical research informat*" OR "Clinical trials informatic*" OR CRI OR "digital care" OR eHealth OR e-health OR etherap* OR "e-Mental health" OR "Health informati*" OR iCBT OR "Internet Cognitive Behavioral Treatment*" OR "medical informatic*" OR mHealth OR m-health OR mtherap* OR m-therap* OR "Online Clinical Trial*")
5	TS=(("clinical research" OR "clinical trial*" OR "medical research" OR health OR intervention* OR psychotherap* OR therap* OR "self-help program*" OR treatment*) NEAR/3 (Computer* OR cyber OR Digital OR electronic OR informatics OR Internet OR Mobile OR Online OR Smartphone OR "Technology Based" OR "Web based"))
6	4 OR 5
7	TS=("design feature*" OR "interaction design*" OR Interface OR "User centered design*" OR "visual* design*" OR usability OR "graphic* design*")
8	3 AND 6 AND 7
9	Filter 2019-2024
